# Galectin-3, a rising star in modulating microglia activation under conditions of neurodegeneration

**DOI:** 10.1038/s41419-022-05058-3

**Published:** 2022-07-20

**Authors:** Juan García-Revilla, Antonio Boza-Serrano, Ana M. Espinosa-Oliva, Manuel Sarmiento Soto, Tomas Deierborg, Rocío Ruiz, Rocío M. de Pablos, Miguel Angel Burguillos, Jose L. Venero

**Affiliations:** 1 Experimental Neuroinflammation Laboratory, Department of Experimental Medical Science, Lund University, BMC B11, Lund, 221 84 Sweden; 2 Departamento de Bioquímica y Biología Molecular, Facultad de Farmacia, Universidad de Sevilla, and Instituto de Biomedicina de Sevilla-Hospital Universitario Virgen del Rocío/CSIC/Universidad de Sevilla, Sevilla, Spain

**Keywords:** Neurodegenerative diseases, Inflammation

## Abstract

The advent of high-throughput single-cell transcriptomic analysis of microglia has revealed different phenotypes that are inherently associated with disease conditions. A common feature of some of these activated phenotypes is the upregulation of galectin-3. Representative examples of these phenotypes include disease-associated microglia (DAM) and white-associated microglia (WAM), whose role(s) in neuroprotection/neurotoxicity is a matter of high interest in the microglia community. In this review, we summarise the main findings that demonstrate the ability of galectin-3 to interact with key pattern recognition receptors, including, among others, TLR4 and TREM2 and the importance of galectin-3 in the regulation of microglia activation. Finally, we discuss increasing evidence supporting the involvement of this lectin in the main neurodegenerative diseases, including Alzheimer’s disease, Parkinson’s disease, Huntington’s disease, amyotrophic lateral sclerosis, multiple sclerosis, traumatic brain injury, and stroke.

## Facts

- Galectin-3 (Gal-3) is a pleiotropic protein that binds to β-galactoside residues present in glycoproteins.
- Gal-3 is mainly expressed and released in the damaged brain by reactive microglia.
- Gal-3 interacts with immune receptors like TREM2 and TLR4.
- Gal-3 appears upregulated in the transcriptomic profile of microglia in distinct neurodegenerative environments.

## Open questions

- Should we consider Gal-3 a broad marker for neurodegenerative diseases?
- Does microglia-secreted Gal-3 play a significant role in non-microglia cells?
- How can the microenvironment affect Gal-3 binding properties and its role in neurodegenerative diseases?
- Could gal-3 inhibitors be a therapeutic option in neurodegenerative diseases?

## A brief overview of galectins

The central nervous system (CNS) is a complex structure of interconnected specialised areas. The functionality of the entire system relies on a delicate equilibrium that is occasionally challenged by the different disrupting stimuli.

This review aims to describe the roles of Galectin-3 (Gal-3) in the diseased brain. Galectins are small proteins that play a variety of functions interacting with glycoproteins and glycolipids from different brain cell types [1]. These promiscuous proteins contribute to the regulation of innate and adaptive immunity, among other processes, and their role depends on their expression levels and preferences for particular structural features of β-galactosides [2].

In general, galectin molecules are based on conserved β-galactoside-binding sites found within their characteristic ∼130 amino acid carbohydrate recognition domains (CRDs) [3]. This conserved domain binds to β-galactosides with different specificities and affinities [4]. Some of these lectins are expressed constitutively, while others are expressed upon stimulation. Importantly, glycosylation patterns change under physiological and pathogenic conditions impacting galectin functions [5].

Among the 15 members of the galectin family, only Gal-1, Gal-3, Gal-4, Gal-8, and Gal-9 have been found to be significantly expressed in the brain, where their functions are still little understood. Galectins are classified according to their CRD into three different groups: ‘Prototypic’ galectins, that have only one CRD that can dimerise (Gal-1, Gal-2, Gal-5, Gal-7, Gal-10, Gal-11, Gal-13, Gal-14, and Gal-15), ‘tandem-repeat’ galectins (Gal-4, Gal-6, Gal8, Gal-9, and Gal-12) with two CRDs within a single polypeptide chain, one at the N-terminal and the other at the C-terminal region, linked by a peptide bridge of variable length. The last group would be the ‘chimera type’ galectin, only represented by Gal-3 bearing a single C-terminal CRD and a non-lectin collagen-like N-terminal region that mediates oligomerisation and regulatory modifications [6].

The immune system contributes to homeostasis by preparing the body to fight infection and help the healing process in the event that harm occurs. Therefore, there must be efficient crosstalk between the different players of the immune response. In this vein, galectins are crucial determinants in neuroinflammatory responses and neuroprotection mechanisms in the brain, recognising glycan structures and sensing their modifications both extracellularly and intracellularly. However, the mechanisms that regulate galectin expression are still unknown. Understanding the functional interplay of different galectins in glial phenotypes and neuronal survival systems under different damaging contexts may reveal new therapeutic possibilities.

## Microglia diversity and Gal-3

Gal-3 is associated with activated microglia since homeostatic microglia do not express Gal-3 [7, 8]. The advent of whole-genome transcriptomic at the single-cell level has revealed a microglia diversity that overcomes the simplistic view of M1/M2 polarisation states under disease conditions [9, 10]. Gal-3 is emerging as a relevant marker in specific clusters associated with activated microglia, as further discussed.

Holtman et al. [11] compared the gene expression networks of microglia isolated from different mouse models of neurodegenerative diseases (Alzheimer’s disease (AD), amyotrophic lateral sclerosis (ALS)) and ageing. They found an independent core gene expression profile that differed from proinflammatory activated microglia [11], including, among other upregulated genes, *Itgax*, *Axl*, *Clec7a*, and *Lgals3* (gene coding Gal-3). In particular, the authors used Weighted Gene Co-expression Network Analysis to identify hub genes likely involved in driving the identified microglia polarisation state under different disease conditions. Following this approach, they identified four hub genes: *Csf1, Axl, Igf1*, and *Lgals3* [11]. Each of these genes was believed to be instrumental in driving essential microglia functions such as proliferation, activation, and phagocytosis. In 2017, two independent studies using single-cell RNA analysis of microglial cells identified a common activated microglia phenotype associated with different brain diseases such as AD, ALS, and multiple sclerosis (MS), which was defined as either disease-associated microglia (DAM) [7] or microglia neurodegenerative phenotype (MGnD) [8]. A common feature of both phenotypes is the downregulation of homeostatic microglial genes such as *P2ry12, Cx3cr1, Hexb* or *Tmem119*, along with upregulation of particular genes, including *Trem2, Apoe, Itgax, Spp1* and *Clec7a* [7, 8]. Intriguingly, Gal-3 was not found to be altered in DAM [7] but highly upregulated in MGnD [8]. However, new studies have clearly shown that Gal-3 is one of the most upregulated genes under conditions of brain disease [12–14]. In fact, the DAM phenotype is exemplified by microglia clustering amyloid beta plaques in AD [7], and we have identified a subset of plaque-associated microglia that express high levels of Gal-3 in human and mouse models of AD [13]. However, new evidence discards a universal common activated microglia phenotype but rather suggests multiple activated subtypes. In fact, distinct and functionally divergent DAM subtypes have been identified in AD mouse models, including proinflammatory and anti-inflammatory profiles [15]. Further evidence is supported by studies by Mathys and colleagues who applied single-cell RNAseq in microglial cells in the CK-p25 inducible mouse of severe neurodegeneration, which develops some aspects of AD pathology [12]. The advantage of using CK-p25 mice is that neurodegeneration occurs shortly after induction of p25, ranging from DNA damage at two weeks to progressive neuronal damage and synaptic loss at six weeks [12]. This short temporal window allows for the characterisation of the molecular signature of isolated microglia at different stages of the disease (early and delayed stages). Using this approach, different microglial clusters were identified after induction of p25, and more importantly, they were identified at different temporal stages (early response versus late response) [12]. Late-response microglia were enriched in a specific cluster (cluster 6), and Gal-3 was identified as one of the most upregulated genes in this cluster [12]. Interestingly, the authors identified genes associated with the antiviral and interferon response and with MHC class II. However, there was no correlation between the antiviral and interferon response modules and the MHC class II module, suggesting the existence of at least two different reactive microglial phenotypes under neurodegeneration conditions. DAM microglia have been suggested to be neuroprotective [7] and MGnD (neurotoxic) [8], and hence the possibility that protective and deleterious reactive microglia populations coexist is certainly plausible. Since Gal-3 deletion has been shown to be neuroprotective in different models of neurodegeneration, including AD [13], it is certainly tempting to speculate that Gal-3 expression makes microglia prone to be neurotoxic. Do we have evidence supporting this view? Two recent studies performed in a model of frontotemporal dementia (FTD) associated with loss of the progranulin (*Grn*) gene support the view that Gal-3 expressing microglia are neurotoxic, at least in this model [16, 17]. The proteomic analysis of Grn knockout mice (Grn KO) identified two proteins, Gpnmb and Gal-3, as two of the most enriched proteins in the brain proteome of Grn KO, especially in aged animals [16]. Both markers were found to be solely expressed by reactive microglia, which were especially evident at sites of damage, including the thalamus, cortex, and hippocampus [16]. Aggregation of the RNA-binding protein TDP-43 in the neuronal cytoplasm and dendrites is a typical hallmark of FTD associated with Grn mutations [18]. Interestingly, a recent study has shown that activated microglia in Grn KO mice act as a key disease driving factor that induces neurodegeneration and aggregation of TDP-43 protein during ageing [17].

It is important to note that DAM is enriched in genes associated with AD pathology. Illustrative examples are *Trem2* and *ApoE*, which play critical roles in the transition from canonical microglia to DAM or MGnD [7, 8]. It is intriguing that a significant number of genes upregulated by DAM are associated with lipid metabolism [7]. In fact, TREM2 has been found to bind to anionic and zwitterionic lipids [19] and regulate myelin debris clearance [20]. However, when TREM2 signalling is disrupted, microglia is prone to acquire a proinflammatory phenotype upon defective lipid metabolism [21]. Even though no direct interaction between Gal-3 and lipid metabolism or myelin has been described, Gal-3 can interact with some membrane lipids [22] anticipating a potential role for Gal-3 in lipid clearance. In that sense, in the cuprizone model of demyelination in WT and TREM2 KO mice, single-cell RNAseq in microglia identified two treatment- and genotype-dependent clusters of activated microglia (clusters 4 and 8). While cluster 4 was predominant in WT mice exposed to cuprizone, cluster 8 was enriched in TREM2 KO [23]. Although genes exclusive to a single cluster were rare, Gal-3 was identified as one of the most exclusive markers of cluster 4 [23], anticipating essential roles for this molecule in driving the microglia response to myelin/cholesterol metabolism in a TREM2-dependent manner. Very recently, Simons and colleagues identified age-dependent white-matter-associated microglia (WAM) that share part of the DAM gene signature and are TREM2-dependent [24]. WAM was enriched in genes related to hypoxia-inducible factor (HIF-1) signalling, lysosomal, and cholesterol pathways along with a robust upregulation of *Clec7a, Axl, Itgax*, and *Lgals3* [24]. WAM has been suggested to play a significant role in the removal of myelin debris most likely associated with degenerated myelin sheaths that accumulate over time during ageing and other neurological conditions [24]. In fact, WAM-like populations were found in mouse models of AD even before the appearance of the DAM signature. Since Gal-3 is a prominent marker of WAM during ageing and in AD mouse models, it remains to be established whether Gal-3 is involved in myelin clearance under disease conditions and during ageing.

It is important to highlight that two independent studies using single-cell RNAseq at different postnatal development stages identified a TREM2-independent microglia subtype associated with white matter [25, 26]. The study by Stevens and colleagues identified a microglial cluster (cluster 4) that was named Axon Tract-Associated microglia (ATM) [25]. ATM showed a very specific spatiotemporal expression (barely detected in adult animals), characterised by expression of high levels of *Spp1, Gpnmb, Igf1, Cd68*, and *Lgals3* and presented amoeboid morphology [25]. Preferential expression included the corpus callosum and cerebellar axon tracts [25]. Following a similar approach, Barres and colleagues identified several microglial clusters on postnatal day 7 [26]. One specific cluster demonstrated high expression of selective genes including *Gpnmb, Spp1, Igf1, Itgax*, and *Clec7a*, and their preferential expression in the corpus callosum and cerebellar white matter [26], thus highly coinciding with the ATM cluster identified by Hammond et al. [25]. This white matter-associated cluster was named proliferative-region-associated microglia (PAM), exhibited an amoeboid morphology again, a feature of high phagocytic function, and was intermingled with Mbp+ oligodendrocytes (OLG) [26]. In fact, PAM exhibited cleaved caspase-3-positive and MBP inclusions, suggesting that this early microglial subtype phagocytose apoptotic newly formed OLG during myelination [26]. Although ATM or PAM share molecular features with WAM, contrary to the last one, (i) the acquisition of the PAM phenotype was not dependent on either TREM2 or APOE, and (ii) while WAM was associated with ageing and neurodegenerative conditions and it is supposed to play a role in myelin clearance, ATM/PAM are transient during early postnatal development, and they are likely associated with phagocytosis of newly formed OLG.

## Role(s) of Gal-3 in microglia activation during neurodegeneration

Our concept of how microglia become activated has changed recently, thanks to the emergence of novel technological advances that substantially increased our knowledge of microglia behaviour under different inflammatory conditions [10]. This has allowed us to evolve from the simplistic classic view of the M1/M2 phenotype to a more comprehensive and realistic point of view [27]. Gal-3 plays an essential role in driving microglial polarisation under different pathological conditions. In the CNS, the interaction of Gal-3 with different proteins (such as different Toll-like receptors (TLRs), Trem2, Igf1R, MerTK, etc.) has been shown to promote a wide variety of responses that could be considered either supportive or detrimental depending on the context. In this section of the review, we will briefly discuss such interactions with Gal-3 and their effect on microglial activation.

In the CNS, one of the most common types of inflammatory response is the pathogen-free type (known as sterile inflammation). This can be promoted by a variety of ligands, including protein aggregates (i.e., α-synuclein (SYN), amyloid β (Aβ)), many of which are known to interact with different TLRs [14, 28–34]. This fact indicates the relevance of TLRs in the progression of different neurodegenerative diseases, such as AD [35]. As mentioned above, in the field of Gal-3 research, crosstalk between Gal-3 and several TLRs [31, 36, 37] has been reported. Functionally, the effect of Gal-3 on the inflammatory response depends on several factors, including the subcellular localisation of Gal-3 [31, 38], the cell type involved [39, 40], or the disease context. For example, reduced expression of Gal-3 reduces TLR-induced IL-6 expression in human synovial fibroblasts [41] and several other proinflammatory cytokines in human monocyte-derived dendritic cells [42]. On the other hand, Gal-3 expression in dendritic cells has been linked to the development of an immunosuppressive environment and an increase in nephrotoxicity in a mouse model with acute kidney injury induced by cisplatin [43].

**Fig. 1:**
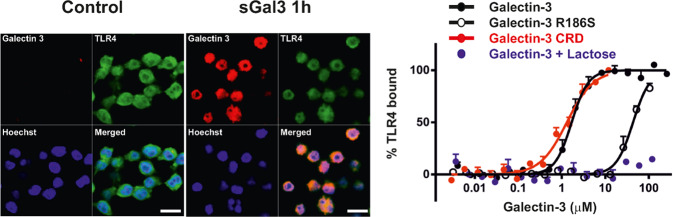
Illustration from Burguillos et al. 2015 [31]: Gal-3 acts as a ligand to TLR4. Left panel: Colocalisation of Gal-3 and TLR4 in BV2 cells after 1 h exposure with sGal-3 protein. Right panel: Microscale thermophoresis was used to analyse the direct binding of TLR4 to Gal-3, Gal-3 CRD, Gal-3 R186S, and Gal-3 in the presence of inhibitory lactose (40 mM). Whereas the concentration of fluorescently labelled TLR4 was kept constant, the non-labelled proteins were titrated (*x* axis), and the minimal and maximal Fnorm values of the unbound and bound state of TLR4, respectively, were used to calculate the percent of TLR4 bound to Gal-3 (*y* axis).

In the CNS, TLRs play a key role in the neuroinflammatory response orchestrated by microglia under different pathological conditions [44]. We have previously demonstrated that Gal-3 mediates the TLR4-induced proinflammatory response under various conditions both in vivo and in vitro (Fig. 1). For example, its absence decreases the proinflammatory response driven by microglia and increases neuronal survival [14, 31, 45]. Interestingly, in two different studies using the Middle Cerebral Artery Occlusion (MCAO) mouse model from the same lab, the authors showed that Gal-3 expression was indeed neuroprotective. In the first study conducted in Gal-3 KO mice, they showed that Gal-3 was capable of interacting with Igf-1R, and it was necessary for proper non-canonical Igf-R1 [46]. This demonstrates the pleiotropic nature of Gal-3 and how the environment can affect Gal-3-receptor interaction and functionality.

Another aspect of the effect of Gal-3 on microglia activation is its ability to initiate the phagocytic response, either as a ligand for MerTK [47] and to promote opsonisation of cells [48, 49] and bacteria [50] or as a ligand for TREM2 [13], mediating the clearance of amyloid plaques. TLRs are also acknowledged to participate in the phagocytic response of latex beads [51], *E. coli* [52], stressed but viable neurons (“phagoptosis” [53]) as well as SYN (in a process termed synucleinphagy [54]). In addition, some studies have shown that TLRs prevent phagocytosis of Aβ [55, 56]. However, a recent study was able to precisely characterise Aβ-phagocytic microglia showing significant upregulation of Gal-3 [57]. The authors identified TLR-pathways as essential for activating such Aβ-phagocytic phenotype in microglial cells. Therefore, based on the previously known ability of Gal-3 to bind several TLRs, it should be taken into account for the potential role of Gal-3 in the phagocytosis of Aβ amyloid plaques.

## Galectin-3 in main neurodegenerative diseases

### Alzheimer’s disease

A growing body of evidence supports the role of inflammatory mechanisms in the development of AD [58–60], and specifically the importance of myeloid cells [61]. Over the last 5 years, Gal-3 has been associated with AD pathology and specifically with microglial activation [8, 13, 62, 63]. Indeed, Gal-3 expression is strictly associated with microglial cell activation around Aβ plaques, and, similar to sTREM2, Gal-3 is released by activated microglial cells under disease conditions [64, 65].

**Fig. 2:**
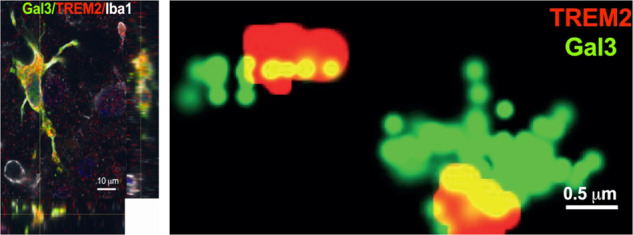
Illustration from Boza-Serrano et al. 2019 [13]: Gal-3 interacts with TREM2. Left panel: Gal-3 and TREM2 in plaque-associated microglia in the brain of 5xFAD mice reveal colocalisation of Gal-3 and TREM2. Right panel: Gal-3 and TREM2 interaction in 5xFAD mouse brain using Stochastic Optical Reconstruction Microscopy (STORM). All images were taken in 5xFAD mice at 18 months.

The brain inflammatory response in AD has focused mainly on DAM [7], which is involved in the inflammatory response and Aβ plaque formation associated with AD and governed by TREM2 signalling [66]. To further unravel the role of Trem2 in AD, Lee et al. used BAC-TREM2 mice to increase TREM2 gene dosage, which induced microglial reprogramming, leading to an overall reduction of AD pathology [67]. Notably, Gal-3 was one of the key microglial genes upregulated in neurodegenerative diseases, along with *Spp1, Gpnmb*, and *Lag* [67], strengthening the relationship between Gal-3 and Trem2. In 2018, we demonstrated upregulation of inflammatory-related pathways in microglial cells prior to plaque deposition (6 weeks of age) in 5xFAD mice [68] using a proteomical approach. In this study, we detected Gal-3^+^ microglia surrounding neurons expressing APP. Later, we were the first to evaluate the role of Gal-3 in AD [13]. The deletion of Gal-3 in 5xFAD mice reduced overall pathology, restored cognitive behaviour, and attenuated microglial immune responses, particularly those associated with TLR and Trem2 signalling, which confirmed the link between Gal-3 and TREM2 [13]. The relation between Gal-3 and Trem2 was further explored by Fluorescent Anisotropy and STORM microscopy. In both cases, we confirmed the binding capacity and proximity of Gal-3 to TREM2, respectively (Fig. 2). Gal-3 was highly upregulated in the brains of AD patients and 5xFAD mice and was mainly associated with Aβ plaques. However, not all microglia around a plaque or near tau-burdened neurons expressed Gal-3, suggesting a specific subset of microglia with a Gal-3-dependent phenotype. We described, for the first time, single-nucleotide polymorphisms associated with the *LGALS3* gene using a meta-analysis approach of several AD cohorts. In particular, a recent large-scale human proteomic analysis of AD brains (>2000 brains) confirmed that the microglia module is one of the most affected in the AD brain and strikingly revealed that the astrocytic/microglial metabolism module was significantly enriched in gene products contained within AD risk factor loci [63]. In that study, the authors identified the 30 top microglial transcripts most differentially abundant in an AD mouse model that overlap with proteins in the human microglia module. Remarkably, within this selected list of candidates, Gal-3 ranked 5th, thus emerging as one of the most promising molecules driving AD pathology [63]. Recently, Monasor et al. identified a large panel of microglial Aβ response proteins (defined as MARPS) involved in microglial activation at different stages of pathology progression in two different AD mouse models, APP/PS1 and APP-KI. Notably, Gal-3 was one of the key proteins upregulated in the early stages of the pathology along with other DAM-associated proteins such as Clec7a, Cd11c, or ApoE [69]. In support of the critical role of Gal-3 in AD pathology, this protein has been measured in the serum of sporadic cases of AD and Mild Cognitive impairment (MCI), showing significantly increased levels in AD cases compared to age-matched controls [70, 71]. Although there is no information published regarding the presence of Gal-3 in microglia from mild cognitive impairment (MCI) postmortem brains, some papers have pointed out the presence of Gal-3 in the serum of these patients, which again suggest a role of Gal-3 in this pathology [70, 72, 73]. Moreover, it has been described that Gal-3 increases in white matter associated with microglia during ageing, which is associated with cognitive impairment. This result suggests that this Gal-3 positive phagocytic microglia could be involved in the processing of accumulating myelin that takes place during ageing [74]. However, despite the importance of Gal-3 in AD, some aspects remain unclear, particularly the role of Gal-3 in tau aggregation and tauopathies where data is scarce. In this context, Lim et al. suggested that Gal-3 could be involved in the removal of aberrant forms of tau by reducing hyperphosphorylation through decrements in the glycogen synthase kinase 3 beta [75].

Finally, using specific Gal-3 inhibitors, we have been able to suppress microglial activation [13, 76]. Inhibitors have been previously used safely in other clinical studies [77] (NCT04473053). Taken together, Gal-3 emerges as an exciting therapeutic target and deserves to be tested clinically to hamper the most common neurodegenerative disease, AD.

### Parkinson’s disease

Parkinson’s disease (PD) is the second most prevalent neurodegenerative disorder in the world. The pathology is characterised by intracytoplasmic accumulation of SYN. Despite the potential role of neuroinflammation in PD, no specific microglial phenotype has been associated with PD. Furthermore, evidence supporting the role of Gal-3 in PD pathogenesis is scarce. We described for the first time the upregulation of Gal-3 and proinflammatory factors in primary microglia in the presence of different forms of SYN [76]. In this study, we demonstrated that phagocytosis of oligomeric and fibrillar forms of SYN provoked the selective overexpression of Gal-3 in vitro and in vivo. Indeed, genetic deletion of Gal-3 or pharmacological inhibition led to reduced inflammatory cytokine production and phagocytic activity. This view was supported by two independent studies showing elevated Gal-3 levels in serum from PD patients [78, 79]. Both studies showed a precise correlation between Gal-3 serum levels and disease progression based on Hoehn and Yahr scores, suggesting a potential role for Gal-3 as a biomarker of PD progression. An increase in Gal-3 levels in serum from PD patients could establish a link between PD and inflammation. For instance, in Garcia-Dominguez et al. [80], we investigated the effect of combined PD and systemic inflammation mouse models. After challenging mice with neurotoxic 1-methyl-4-phenyl-1,2,3,6-tetrahydropyridine (MPTP) and proinflammatory lipopolysaccharide (LPS), we observed increased neurodegeneration and cytokine release and, more importantly, the appearance of selective Gal-3 positive microglia in the substantia nigra of mice after combined treatment with MPTP and LPS. Similarly, Gal-3 has been described to increase after MPTP treatment, but only in a transient manner after the first hours [81]. Interestingly, this increase was abrogated in TREM2 knockout mice, suggesting that a microglia PD-specific phenotype reminiscent of DAM microglia is plausible. That would place Gal-3 on the verge between inflammation and neurodegeneration (or vice versa); however, other authors have shown that Gal-3 can play alternative roles in PD beyond neuroinflammation. For instance, Flavin and colleagues identified Gal-3 in the outer layers of Lewy Bodies from PD patients, which they associated with increased vesicle rupture [82]. To our knowledge, this is the first demonstration of intraneuronal Gal-3 in the context of PD. The authors also showed that Gal-3 selectively binds to lysosomes after SYN fibrils stimulation in vitro, promoting autophagy induction of broken lysosomes. Gal-3 is known to bind to broken lysosomes (and other organelle membranes) and participate in mechanisms that try to repair the damaged vesicle interacting with the ALG-2-interacting protein X (ALIX) and possibly with the leucine-rich repeat kinase 2 (LRRK2) [83, 84]. At the same time, Lewy Bodies are structures that can be full of damaged organelles, particularly lysosomes and mitochondria [85]. These studies suggest that Gal-3 plays a significant role in the dynamics of SYN within the affected neuron, particularly in lysosome and organelles damage. It should be considered that microglia can release Gal-3 into the extracellular space [62], which could be a source of Gal-3 from neighbouring neurons and also related to lysosome damage and organelle recruitment to the outer layer of Lewy Bodies. These studies raise the exciting view that Gal-3 may be involved in Lewy Body formation and/or neurotoxicity.

### Gal-3 and Huntington’s disease

Huntington’s disease (HD) is an autosomal dominant degenerative motor disorder that manifests itself with movement dysfunction. It is caused by CAG repeats in the Huntingtin (HTT) gene [86]. When they are more than 36 years old, the mutant HTT protein (mHTT) forms inclusions that compromise brain cell functions [87]. Previous studies have suggested that irregular activation of microglia contributes to the pathogenesis of HD [88, 89]. In this sense, as in other neurodegenerative and neuroinflammatory disorders, Gal-3 has recently been described to play an essential role in brain inflammation associated with HD [90]. In this study, the authors observed that the plasma levels of Gal-3 increased in HD patients. Furthermore, these levels were notably correlated with the severity of the disease. At the brain level, upregulation of Gal-3 was found in the caudate putamen region of the postmortem brains of these patients [90].

In animal models, R6/2 mice (mHTT transgenic mice) presented elevated plasma Gal-3 levels in 12-week-old mice. Furthermore, R6/2 mice presented elevated Gal-3 levels in the striatum, resembling what has been observed in patients. This upregulation was only detected in microglia, occurred before motor impairment, and remained throughout disease progression to the end stage. In HdH150Q mice (a knock-in model), increased levels of Gal-3 protein and transcript were also found [90]. Interestingly, Gal-3 was also expressed in some microglia from aged WT mice. Thus, ageing could contribute to the upregulation of Gal-3 [74].

An important mediator of Gal-3 regulation in HD microglia appears to be Nuclear factor κB (NFκB). It has been demonstrated that NFκB inhibition in primary microglia culture reduces the expression of Gal-3, decreasing microglia activation and the production of proinflammatory cytokines [90]. Nevertheless, at the same time, Gal-3 upregulation is necessary for NFκB abnormal activation and subsequent inflammatory response in HD microglia, indicating a positive feedback loop between NFκB-Gal-3 in microglia. This regulation of the inflammatory response by Gal-3 could be through an intracellular site, without compromising TLR4 [62], being irrelevant the level of extracellular Gal-3 to the status of microglia activation [90]. Interestingly, the same study suggested that Gal-3 could promote the assembly of the NLRP3 inflammasome triggering neuroinflammation [90].

Similarly to what is observed in PD, mHTT has been shown to induce vesicle rupture in SH-SY5Y cells, as observed by the formation of Gal-3 puncta [82]. Moreover, upregulated Gal-3 has been found to form puncta in damaged lysosomes of HD microglia, interfering with clearance and contributing to overactivation of the neuroinflammatory response [90].

To dig into the role of Gal-3, Siew et al. also used a knockdown strategy. The results obtained in HD mice showed that Gal-3 suppression improves microglia-mediated pathogenesis [90]. All these results suggest Gal-3 as a novel target for the development of therapeutic treatments for HD.

### Amyotrophic lateral sclerosis (ALS)

Neuroinflammation has been described in motor neuron disease, including ALS and spinal muscular atrophy (SMA) diseases in murine models (for a review, see [91, 92]). Galectin 3 has been mainly described in ALS. In fact, an increase in Gal-3 expression has been observed in motor neurons and muscle cells [93, 94], proposing Gal-3 as a candidate biomarker for ALS [95], which correlates preferentially with microglial activation in the region where motor neurons degenerate [96, 97]. Interestingly, DAM microglia have also been described in the spinal cord of ALS mice models [7], including an important upregulation of Gal-3. Furthermore, Lerman et al. observed an increase in Gal-3 expression in the spinal cord of sporadic ALS patients, specifically in microglia [98]. In the same study, the authors observed that, unlike what was observed with other models of neurodegeneration (for example, in AD), the elimination of Gal-3 expression in SOD1^G93A^ ALS mice made the disease progress faster. In addition, although the onset was similar, mice died on average 25 days earlier than SOD1 ^G93A^/Gal-3^+/+^. Therefore, the presence of Gal-3 in the regions of the spinal cord where motor neuron death occurs could be protective as the disease progresses worse by eliminating Gal-3. ALS undetermined pathogenesis makes it difficult to explain the reported protective effect of Gal-3 in this disease compared with other neurodegenerative diseases. However, the reason could be due to distinct roles of Gal-3 beyond microglia activation like differentiation of oligodendrocytes (see Multiple Sclerosis section), Schwan cells [99] or neuronal lysosomal repair [100] that could overall counteract the deleterious inflammatory effect. A similar experimental approach should be carried out in alternative models of ALS to confirm this deleterious effect when Gal-3 is eliminated.

### Multiple sclerosis

Multiple sclerosis (MS) is an autoimmune disease characterised by the loss of myelin in the nerves, which leads to poor conduction of action potentials and, therefore, synaptic dysfunction [101]. Oligodendrocytes are responsible for wrapping nerves with myelin and supporting the axon. They are generated from oligodendrocyte progenitor cells that arise from the subventricular zone (SVZ) [102]. In fact, oligodendroglial injury triggers demyelination, which is followed by a remyelination process that forms a new myelin sheet [103, 104]. The roles of Gal-3 in CNS myelinisation and remyelination have been deeply described (for a review, see [2]), including its key role in oligodendrocyte differentiation [105, 106] and proliferation [107]. In addition, the involvement of Gal-3 in demyelinating diseases was established when an increase in its expression was observed in active injured regions in MS patients [108]. Furthermore, the presence of Gal-3 autoantibodies in serum from MS patients [109] and Gal-3 upregulation in postmortem MS human brain tissues has been described [110].

The most widely used animal model that mimics CNS demyelination is experimental autoimmune encephalomyelitis (EAE), which comprises immunised mice with myelin oligodendroglial glycoprotein. The lack of Gal-3 in the EAE model reduced the severity of the pathology and macrophage infiltration [111]. This study suggested a fundamental role for Gal-3 in the recruitment of leukocytes during the inflammatory process. In fact, Itabashi et al. showed that Gal-3 expression changed during the progression and recovery of EAE, suggesting a neuroprotective role in EAE mice [112]. Supporting the beneficial role of Gal-3 in EAE, the absence of Gal-3 in a virus-induced demyelination model decreased the number of immune cells in the SVZ and restored proliferation [113].

The cuprizone-induced demyelinating model (CPZ) has also been used for the study of MS. This model leads to intense demyelination of the corpus callosum and activates progenitor cells in the adjacent SVZ. It is characterised by microglial recruitment in the damaged area, while the role of peripheral macrophage infiltration remains limited [114, 115]. In 2016, Hoyos et al. investigated the role of Gal-3 in this model and found that Gal-3 played a key role in the control of the microglia response [116]. Thus, the absence of Gal-3 expression impaired the ability of remyelination. The authors described that Gal-3 expression increased during CPZ-induced demyelination in microglia, and when Gal-3 expression was abolished, microglia located in the demyelination / redemyelination zone showed decreased levels of activation markers such as Cd68 and Trem2. Therefore, these results indicate that Gal-3 could have a potential role as a modulator of microglial activation in the CPZ model [106, 116, 117]. In contrast, Hillis et al. showed that Gal-3 normally limited SVZ cell emigration following CPZ treatment but failed to find any effect of Gal-3 deletion in myelin loss or remyelination in the corpus callosum [118]. They attributed this discrepancy to using different Gal-3 knockout mice that present different exons deletion. For example, the mouse used by Hoyos et al. lacks exon V, which encodes one crucial part of the CRD domain [119], but the N-terminal domain of the protein could remain intact. This highlights the possibility of CRD-dependent and independent roles for Gal-3 in MS. Finally, because the function of Gal-3 differs from cell to cell and plays a key role in the phagocytosis of myelin debris and the differentiation and proliferation of OLG, it would be interesting to know the exact mechanism and through which the cell type Gal-3 exerts the neuroprotective or detrimental role. Therefore, further experiments are required to gain deep insight into this question.

### Traumatic brain injury

Traumatic brain injury (TBI) is one of the leading causes of death and disability in the western world. TBI pathology may result in a complex set of symptoms that may lead to long-lasting impaired cognitive function and dementia, PD, or ALS [120]. Rapid actions in both the pre-hospital and early in-hospital stay are considered key components to decrease mortality and improve the neurological outcome of the patient [121].

**Fig. 3:**
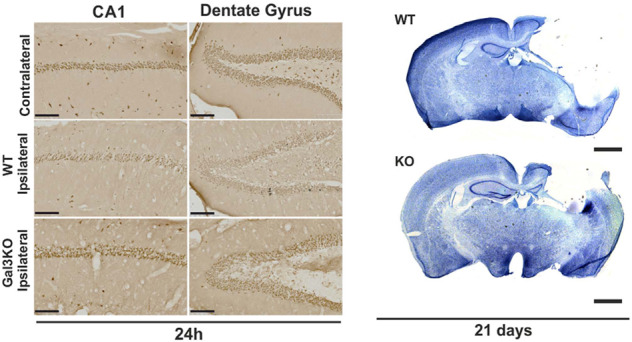
Illustration from Yip et al. 2017 [14]: effect of the lack of galectin-3 on the neuronal survival in cortex and hippocampus after TBI. Representative pictures of NeuN in different regions (left panel). Representative image of toluidine blue staining in wild-type and galectin-3 knockout mice (right panel). Note neuroprotection at 24 h and limited injury size 21 days after the TBI in Gal-3 KO mice. Scale bars are 100 and 1000 µm respectively.

It is known that under head trauma conditions, using different trauma models, including spinal cord injury [122–124] and experimental models of TBI [125, 126], there are striking early increases in Gal-3 expression. In fact, there is not only an increase in the expression of Gal-3 (mainly in microglia) but also an increase in its release in CSF in vivo [14] and in plasma from patients with TBI [127, 128]. Recently, it has been established a positive correlation between Gal-3 levels in plasma and Glasgow Coma Scale scores [127], suggesting that Gal-3 could be a potential biomarker for TBI. To determine the role of Gal-3 in TBI, we administered a neutralising antibody against Gal-3 one hour after head injury. We observed that Gal-3 neutralisation conferred neuroprotection in the cortex and hippocampal cell populations (Fig. 3) and decreased expression of IL-1β, IL-6 and NOS2 [14]. Due to the importance of TLR2 and TLR4 in TBI-associated neuroinflammation [129, 130], we studied the Gal-3/TLR4 interaction and found that, after a head injury, Gal-3 was immunoprecipitated with TLR4 [14]. The latest suggests that either blocking or inhibiting released Gal-3 could be a possible therapeutic treatment against TBI.

### Stroke

Ischaemic stroke can be caused by vasoconstriction, embolism, or thrombosis. In this situation, microglial cells become activated and are recruited to the site of the injury leading to neuronal cell death [131]. Therefore, strategies aimed at regulating the detrimental inflammatory response could be an attractive alternative to treat this condition.

Several experiments in animal models have studied the potential role of Gal-3 in modulating glial responses after different types of stroke models. For example, Gal-3 levels are known to increase in the perihematomal brain region in an intracerebral haemorrhage model (a devastating type of stroke) from day 3 to day 7 after injury. In these conditions, Gal-3 is expressed primarily in proinflammatory microglial cells [132]. Indeed, we and other authors have demonstrated that Gal-3 is necessary for the activation and proliferation of microglial cells after an ischaemic injury [133]. This effect is related to the Gal-3/Igf-R1 interaction following an ischaemic injury [46]. We demonstrate the crucial role of Gal-3 and the Gal-3/TLR4 axis in the associated neuroinflammatory response and neurodegeneration. Genetic deletion of Gal-3 leads to lower cytokine release and protection against neurodegeneration 8 days after ischaemia. Importantly, we confirmed this important axis in microglial cells from human stroke brain samples [31]. However, some studies have shown discrepancies about the role of Gal-3 in stroke. For instance, 24 h after MCAO, intracerebroventricular injection of recombinant Gal-3 induces downregulation of proinflammatory cytokines and upregulation of anti-inflammatory cytokines, thus leading to a therapeutic shift in microglial polarisation that was associated with a reduction in the infarct size [134]. As we discussed previously, these seemingly contradictory roles may be due to differences in the time of artery occlusion [31, 46, 134].

Interestingly, Gal-3 positive microglial cells emerge after the onset of neuronal cell damage after transient ischaemia [135]. Similarly, we have previously demonstrated that Gal-3 is upregulated after a mouse model of global brain ischaemia [31]. Chip et al. demonstrated that genetic deletion of Gal-3 enhances neuroinflammation 72 h after a transient MCAO in postnatal mice [136]. Furthermore, downregulation of Gal-3 using siRNA increases neuronal viability while decreasing proinflammatory cytokine expression levels in vitro [137]. Similar results were found in a model of neonatal hypoxic-ischaemic brain injury, including a mild reduction in neuroinflammation in Gal-3 knockout mice [138]. Gal-3 is also known to be necessary for angiogenesis in stroke in a manner dependent on vascular endothelial growth factor (VEGF) [139]. The protective roles of Gal-3 against ischaemic stroke also seem to be mediated via apoptosis inhibition through Akt/Caspase regulation [140]. Finally, Gal-3 can activate Ca^2+^ signalling, another important mediator in inflammation that follows a stroke. The induction of Ca^2+^ influx activates protein kinase C and subsequently induces IL-4 expression [141].

For all these reasons, it is not surprising that blocking Gal-3 using neutralising antibodies decreases angiogenesis and proliferation of neuronal progenitors, suggesting that Gal-3 could play an important role in postischemic tissue remodelling by enhancing angiogenesis and neurogenesis [133] as well as inducing an anti-inflammatory phenotype on microglial cells.

Several studies have proposed serum levels of Gal-3 as a biomarker of the severity and prognosis of stroke in patients [137, 142–146]. The most predictive value of serum Gal-3 levels after a stroke occurs when this measurement is performed at the time of admission [147] in particular in combination with the measurement of N-terminal pro-brain natriuretic peptide levels (NT-proBNP; [148]) and high-density lipoprotein (HDL) cholesterol [149].

Furthermore, some compounds with protective effects on stroke, such as the Chinese medicine QiShenYiQi, melatonin, and modified citrus pectin (MCP), exert their effects through decreased expression of Gal-3 [149, 150] or its inhibition [151]. In keeping with this view, in 2020, a preliminary study has demonstrated that the administration of the Gal-3 inhibitor MCP ameliorates brain oedema and neuronal score in an experimental mouse model of subarachnoid haemorrhage [152].

All these data support the study of the role of Gal-3 in stroke and the possible therapeutic strategies aimed at improving the outcome of this severe disease based on the regulation of the expression or release of Gal-3.

## Concluding remarks

**Fig. 4:**
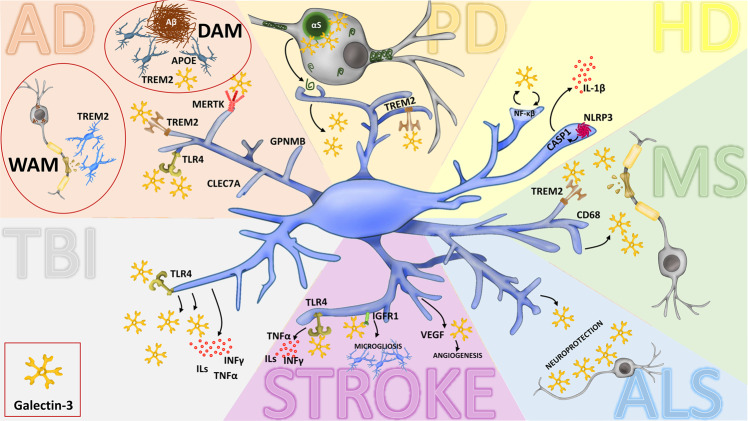
Broad view of the role of Galectin-3 in microglia-associated neurodegenerative diseases. Galectin-3 (Gal-3) is one of the most highly expressed in Alzheimer’s Disease (AD) associated microglia (DAM) interacting with TREM2, TLR4 and MERTK. White matter-associated microglia (WAM) is also triggered by TREM2 activity but no role for Gal-3 has been yet uncovered. Gal-3 is found in the outer layer of the Lewy Bodies in neurons from Parkinson’s Disease (PD) patients, whereas internalisation of alpha-synuclein (αS) by microglia leads to the release of Gal-3 to the extracellular matrix. In Huntington’s Disease (HD) Gal-3 is known to be released by microglia leading to inflammasome pathway activation and cytokine release. In Multiple Sclerosis (MS) Gal-3 is released in response to demyelinization and known to interact with TREM2. In Amyotrophic Lateral Sclerosis (ALS) microglia release Gal-3 which exerts a neuroprotective role, but no mechanism nor ligands have been identified. In Stroke, Gal-3 present a dual role binding to TLR4 and IGF1R to increase cytokine production and microgliosis, but also promoting VEGF release and angiogenesis. In Traumatic Brain Injury (TBI), Gal-3 is highly released after the trauma and binds to TLR4 predominantly to promote an intense inflammatory reaction associated to cytokine release.

Different activated microglia subtypes highly express Gal-3, making this lectin an interesting candidate to drive microglia-associated immune responses under disease conditions. The pleiotropic roles of Gal-3 are inherently associated with its ability to interact with different microglial receptors, including TLR4, TREM2, MerTK, etc (Fig. 4). Each of these receptors is a key component of the microglia response to DAMPS. Illustrative examples include Aβ and SYN, which are believed to drive the main activated microglia phenotypes under conditions of brain disease. For instance, TLR4 drives a microglia proinflammatory phenotype, while TREM2 is fundamental in driving either the DAM/MGnD or WAM subtypes (Fig. 4).

The potential involvement of Gal-3 in the pathology associated with aged-related, familial and acute neurodegenerative diseases is exemplified by increased serum or CSF levels of this lectin in AD, PD, ALS, TBI, and stroke, thus increasing its utility as a potential biomarker in disease progression. Since the expression of Gal-3 in the diseased brain occurs primarily in activated microglial cells, a major challenge for the scientific community will be identifying neurotoxic microglial cells in the different diseases and how Gal-3 contributes to the switch from homeostatic to neuroprotective/deleterious phenotypes.

## Data Availability

Data sharing not applicable to this article as no datasets were generated or analysed during the current study.
